# A Subserosal Fibroid With Myxoid Degeneration: A Challenging Pelvic Mass

**DOI:** 10.1155/crog/3469082

**Published:** 2026-08-11

**Authors:** Bassem Skaff, Christopher Massaad, Kariman Ghazal

**Affiliations:** ^1^ Department of Obstetrics and Gynecology, New Mazloum Hospital, Tripoli, Lebanon; ^2^ Faculty of Medicine, Saint George University of Beirut, Rmeil, Lebanon; ^3^ Department of Obstetrics and Gynecology, Faculty of Medical Sciences, Lebanese University, Hadath, Lebanon, ul.edu.lb

**Keywords:** laparoscopy, leiomyoma, myxoid degeneration, pelvic mass

## Abstract

**Background:**

Leiomyomas are common gynecological masses. However, atypical variants, such as those with myxoid degeneration, can mimic malignancies. This, in turn, might result in diagnostic challenges.

**Case Presentation:**

A 29‐year‐old female presented with chronic pelvic pain and backache. Imaging revealed a 10‐cm adnexal mass with features suspicious of endometrioma or malignancy. Laparoscopic resection was performed. Histopathology confirmed a leiomyoma with myxoid degeneration.

**Discussion:**

Myxoid degeneration, a rare type of leiomyoma degeneration, can alter typical findings on imaging. It can also lead to uncertainties when it comes to diagnosis. This case highlights the importance of correlating imaging with histopathology to avoid unnecessary or radical surgeries.

**Conclusion:**

Atypical leiomyomas should be considered in the differential diagnosis of pelvic masses, despite their rarity. Accurate diagnosis and management can prevent overtreatment. It also ensures optimal patient outcomes.

## 1. Introduction

Leiomyomas are common gynecological masses in reproductive‐aged women, with a prevalence ranging between 20% and 40% [1]. Although leiomyomas can be well diagnosed with ultrasound, atypical variants can exist. The latter can display different imaging characteristics that may lead to misdiagnosis [2, 3]. Unusual manifestations of leiomyomas comprise degeneration, internal changes, unusual pattern, and extrauterine growth. Degenerative patterns of leiomyomas include hyaline (> 60%) [3], cystic, calcified, red, myxoid, and, rarely, sarcomatous change (0.1%–0.8% of leiomyoma specimens) [4]. Specifically, myxoid degeneration is characterized by a benign and rapidly growing mass. It comprises 1%–3% of all degenerative types, with a histological appearance of mucoid areas [4].

These changes alter the usual characteristics of leiomyomas on imaging, which in turn can lead to uncertainty in diagnosis and eventually misdiagnosis or overtreatment [5, 6]. From this perspective, histopathology remains crucial for the definitive diagnosis of challenging pelvic masses [3].

We present this case to illustrate the diagnostic pitfalls of myxoid leiomyoma degeneration with the aim of raising awareness of this rare entity among clinicians managing atypical pelvic masses.

## 2. Case Presentation

A 29‐year‐old nonsexually active woman presented with chronic pelvic pain, back pain, and pelvic heaviness of several months′ duration. The patient′s symptoms were gradually worsening and were associated with difficulty defecating. Her menstrual cycles were irregular. The patient reported heavy flow but no dysmenorrhea. Vitals were within normal limits. Physical examination showed abdominal distension with mild lower quadrant tenderness. Her hemoglobin was within the normal range, without evidence of anemia. Tumor markers were within normal limits except for a mildly elevated CA‐125 of 87.8 units/mL.

**Figure 1:**
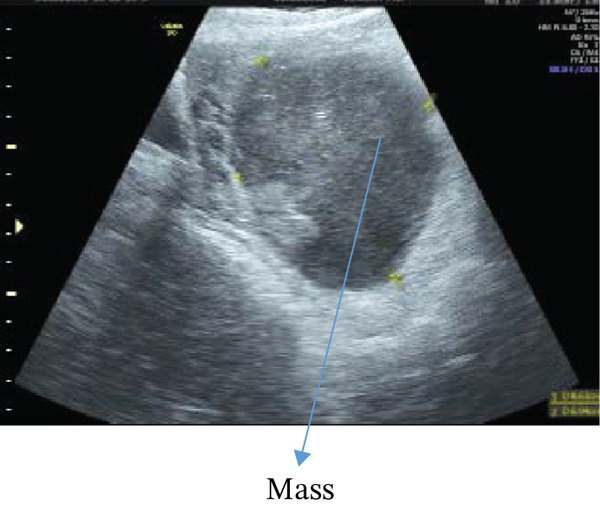
Homogeneous well‐circumscribed adnexal mass with ground‐glass appearance of endometrioma.

**Figure 2:**
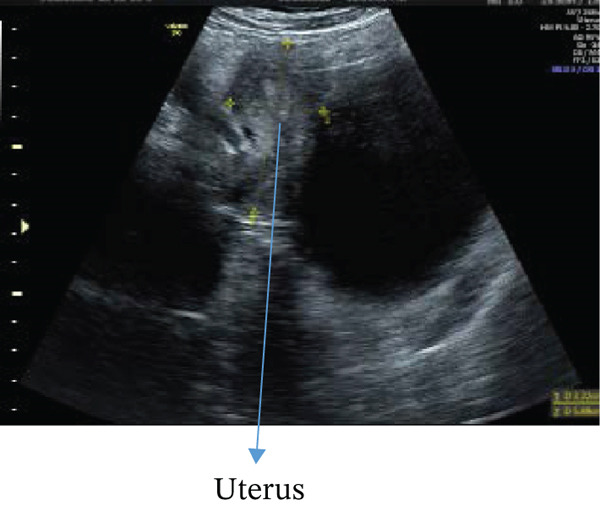
Normal looking uterus.

**Figure 3:**
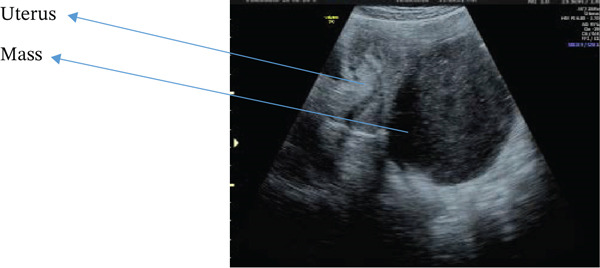
A giant 9‐cm right homogenous ovarian cyst with a thick lining of 80 mm.

**Figure 4:**
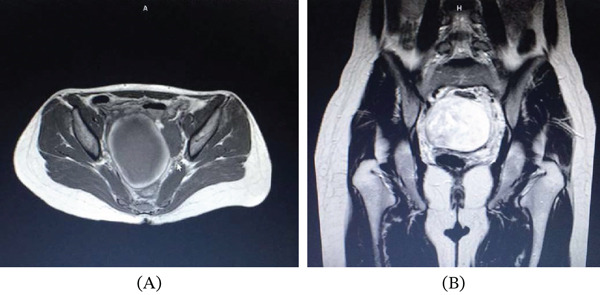
(A) Transverse and (B) coronal view: contrast MRI of the pelvis showing 10‐cm adnexal mass suspicious of right cystadenoma of the ovary with no lymphadenopathies or other notable pelvic lesions.

Three independent ultrasounds consistently favored an ovarian or adnexal cystic lesion. A 9‐ to 10‐cm right‐sided, homogenous cystic mass (Figures 1 and 2) with a thickened wall (0.8 cm) was noted. One assessment noted mild fluid in the pouch of Douglas (Figure 3). Another supported a chocolate cyst with a regular contour and no Doppler vascularity. MRI with gadolinium was done (Figure 4). Likewise, it favored a right ovarian cystadenoma. A well‐defined 10‐cm lesion, hypointense on T1 and hyperintense on T2 was noted. It had peripheral rim enhancement and no internal enhancement, lymphadenopathy, or invasion of adjacent structures.

**Figure 5:**
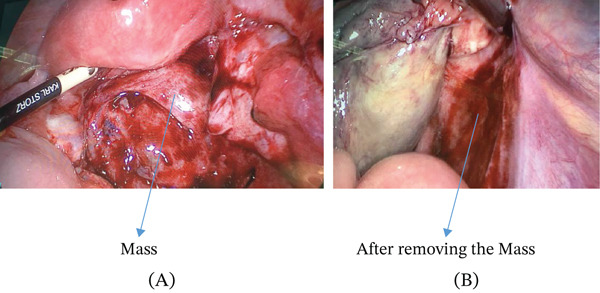
(A, B) Intraoperative photos of the pelvic mass with thick wall and inflammatory abdominal wall changes.

**Figure 6:**
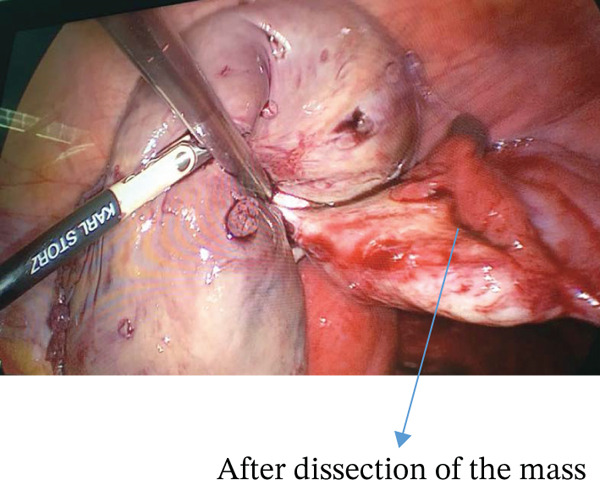
Pelvic area after dissection of the mass.

**Figure 7:**
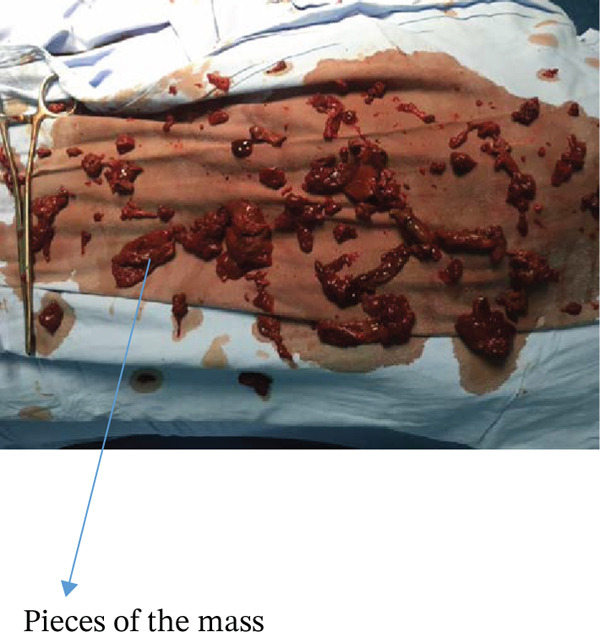
Gross visualization of the mass after its removal by the morcellation bag.

Laparoscopy revealed a large right‐sided mass with a thick capsule. The latter found loosely adherent to the bowel, cul‐de‐sac, uterus, and right ovarian fossa with a diffuse inflammatory reaction (Figure 5). The mass arose from the posterolateral aspect of the uterus with subserosal origin. The right ovary displaced beneath it. Dissection was carried out using suction and atraumatic forceps. The estimated blood loss was minimal. The right ovary was masked underneath the mass, which was completely resected (Figure 6). The mass was completely resected and removed within a morcellation sac through the left accessory port (Figure 7). The patient had an uneventful postoperative recovery. Gross pathology revealed a well‐circumscribed, tan‐white mass with a gelatinous, mucoid cut surface. Microscopically, the lesion showed spindle cell fascicles within abundant myxoid stroma and low cellularity. No significant nuclear atypia and no coagulative tumor necrosis were noted. The final pathology confirmed a leiomyoma with myxoid degeneration, which was initially not reflected on imaging.

## 3. Discussion

The differential diagnosis of a pelvic mass varies widely between infectious, benign, and malignant conditions. Indeed, there are inherent limitations of scoring systems such as ROMA, OVA1, RMI, and imaging modalities in distinguishing benign from malignant pelvic masses. Therefore, careful clinical reasoning should be correlated with histopathological findings for definitive diagnosis [7, 8].

On ultrasound, uterine leiomyomas typically appear as well‐defined, hypoechoic solid masses with peripheral vascularity on Doppler [9]. Ultrasound findings depend on the type of degeneration present. Indeed, hyaline degeneration may produce heterogeneous echogenicity. Calcified variants often show acoustic shadowing. Cystic or myxoid degeneration produces anechoic or pseudocystic areas that can closely mimic an ovarian endometrioma, mucinous cystadenoma, or even a malignant mass [10, 11].

In our patient, this atypical presentation was borne out in practice. Indeed, three independent ultrasound examinations consistently favored an ovarian or adnexal cystic lesion (endometrioma, cystadenoma, or a chocolate cyst with a thickened wall) rather than the classic hypoechoic, well‐defined solid appearance of a leiomyoma. This discordance between the true uterine origin of the mass and its cystic sonographic appearance was the central driver of the diagnostic uncertainty in this case. This further needed evaluation with MRI.

Moreover, it is worth noting that preoperative imaging can also be challenging. Indeed, a high‐T2 signal rim sign can be present in up to 23% of uterine leiomyomas [12]. This is also the case of myxoid leiomyosarcomas, which can demonstrate high‐signal intensity on T2‐weighted MRI [13, 14]. These features can also overlap with malignant pelvic tumors [14]. On another level, the pedicle sign—which differentiates pedunculated subserosal leiomyomas from adnexal masses—was not identified in ultrasounds done. When it is identified, it points out to a direct vascular connection between the mass and the myometrium. However, as demonstrated in our case, the pedicle sign may not always be detectable.

Studies on myxoid degeneration of fibroids are limited and predominantly consist of case reports. In our case, ultrasonography mimicked an endometrioma while MRI favored malignancy over a benign condition. Kamra [15] reported a cervical mass in pregnancy. The latter was managed with intrapartum excision that proved to be a fibroid undergoing myxoid degeneration. Indeed, as in our case, the correct diagnosis was reached only after histopathology. AlShalabi et al. [16] reported a 17 × 12 × 10‐cm extrauterine myxoid leiomyoma in a perimenopausal woman, which was managed by excision. This further shows that, similarly, in our patient, extrauterine or subserosal location further complicates preoperative imaging assessment. In the case of Panda et al. [5], a large uterine mass with nonspecific imaging characteristics underwent exploratory laparotomy. Frozen section biopsy preliminarily suggested a myxoid mass with sarcoma not excluded. In their reported case, TAHBSO was performed, but final pathology documented no atypia. This further reinforces that even intraoperative frozen section can be misleading. Therefore, histopathology remains essential before committing to radical surgery. Similar reports of inconclusive imaging with suspicion of malignancy may lead to more extensive surgery, such as TAHBSO [17]. Similar to our case, Ovayolu et al. [6] documented a huge adnexal mass suspected to be ovarian cancer, for which TAHBSO was also performed. The latter proved to be myxoid degeneration of a fibroid. Relative to this existing literature, our case is distinguished by its laparoscopic, rather than open, management. This is despite having a 10‐cm mass with dense adhesions. This further supports the feasibility of a minimally invasive approach even when imaging is highly suspicious for malignancy.

In summary, this case underscores that myxoid degeneration of a leiomyoma should be considered in the differential diagnosis of atypical or inconclusive pelvic masses. This should be the case particularly when imaging findings are ambiguous or raise suspicion for malignancy. Clinicians should be aware that imaging, including MRI, may remain inconclusive in such cases. Histopathology remains the gold standard for definitive diagnosis.

## 4. Limitations

This article is limited to reporting one single case of subserosal fibroid with myxoid degeneration, which further restricts the generalization of our findings. In addition, with the absence of long‐term follow‐up, it remains difficult to assess for delayed complications or recurrence.

## 5. Conclusion

This case illustrates that a subserosal leiomyoma with myxoid degeneration can closely mimic an adnexal malignancy on both ultrasound and MRI. It also showcases that even with a mildly elevated tumor marker and with imaging suggestive of malignancy, clinicians should not preclude consideration of a degenerating leiomyoma in the differential diagnosis. Histopathological confirmation remains the gold standard for diagnosis. Accurate diagnosis can prevent overtreatment while ensuring optimal patient outcomes.

## Author Contributions

Dr. Kariman Ghazal and Dr. Bassem Skaff spearheaded this case report encompassing the conception and design, data acquisition, analysis, and interpretation. They played a pivotal role in drafting the manuscript and provided final approval for the version to be submitted. Dr. Christopher Massaad further enriched the manuscript by critically revising it for significant intellectual content and also extended their approval for the final version to be submitted. Dr. Kariman Ghazal approved the final version of the manuscript while also approving the technique used in the surgery. The study was designed, implemented, written, and presented for publication by Dr. Kariman Ghazal

## Funding

No funding was received for this manuscript.

## Ethics Statement

Ethical approval to report this case was obtained from New Mazloum Hospital by Dr. Bassem Skaff (chairman). However, due to the ongoing crisis in Beirut, Lebanon, the ethical approval number is unavailable. Despite the challenges posed by the current situation, all relevant rules and guidelines for conducting and reporting medical case studies were strictly adhered to. Patient confidentiality was maintained, and informed consent was obtained from the patient for the publication of this case report and any accompanying images. The hospital′s ethics committee reviewed the case to ensure that it met ethical standards for clinical reporting.

## Consent

Written informed consent was obtained from the patient(s) for their anonymized information to be published in this article.

## Conflicts of Interest

The authors declare no conflicts of interest.

## Data Availability

The data that support the findings of this study are available from the corresponding author upon reasonable request.
